# Japanese encephalitis: a review of clinical guidelines and vaccine availability in Asia

**DOI:** 10.1186/s40794-015-0013-6

**Published:** 2015-11-09

**Authors:** Patricia Batchelor, Kyle Petersen

**Affiliations:** 1 Australian Federal Police Medical Services, GPO Box 401, Canberra City, ACT 2601 Australia; 2 Uniformed Services University of the Health Sciences, 4301 Jones Bridge Road, Bethesda, MD 20814-4799 USA

## Abstract

Travelers to Asia are at risk for acquiring Japanese Encephalitis (JEV), an arbovirus with high rates of morbidity and mortality. Recent advances in vaccination resulting in vaccines with low rates of side effects have strengthened the rationale to vaccinate more travelers to this region, as reflected in many updated national guidelines for prevention of disease in travelers. Vaccines however still require a complex pre-travel schedule and are costly, often leading to a requirement or desire for a vaccination option in the destination country. We explore current national guidelines for prevention of Japanese Encephalitis and seek to provide information on availability of JEV vaccines in various Asian countries.

## Introduction

Japanese encephalitis virus, a flavivirus transmitted by mosquitoes, is the leading cause of encephalitis in Asia [1]. The Japanese encephalitis (JE) cycle occurs between mosquitoes (mainly the species *Culex tritaeniorrhynchus*) and vertebrates. Swine and waterfowl play an important role as amplifying hosts [2]. Humans are a dead end host and as such there is no human-to-human transmission. This mosquito species preferentially breeds in rice paddies and is a dusk and evening feeder. Approximately 99 % of JE infections are asymptomatic; however, JE in symptomatic patients can be a devastating disease with a mortality of approximately 30 %. Following an incubation period of 4–14 days, symptomatic patients can present with high fever, chills, headache, myalgia and confusion. In children gastrointestinal complaints (pain and vomiting) can dominate initially and 75 % will experience seizures [1]. Up to 50 % of survivors will have persistent neurological deficits and this is particularly important in children as there may ensue a lifetime of handicap. Children and the elderly are more at risk of infection [3]. No treatment is available and thus apart from mosquito bite avoidance, vaccination is the only reliable method of prevention. Unfortunately in most countries a multiple injection series of vaccinations is required for pre-travel prevention. Many travellers fail to anticipate this and arrive at a clinic with inadequate time to achieve complete immunity prior to travel. However, as many patients who are at risk of Japanese Encephalitis have itineraries lasting several weeks to years, an opportunity to vaccinate the patient during travel exists that can mitigate the timing difficulties mentioned above. Hence, a comprehensive list of available vaccines by country and providers by city would be of value to many travel clinic providers, who could then counsel their patients on options for vaccination upon arrival to Asia. We will review the various current guidelines for vaccination and provide information about availability of various vaccines in Asia as an aid to the travel medicine provider.

## Review

The World Health Organisation reported an estimated 67 900 cases of JE in 2012, however this is likely to be an underestimate, as reporting depends on the ability to clinically and serologically diagnose the disease as well as the availability of surveillance systems [4]. Furthermore the infectious burden may be vastly underestimated as the ratio of asymptomatic to symptomatic infections is estimated to be between 1:25 and 1:300 [5].

Two patterns of transmission are recognised – epidemic and endemic. In Northern areas such as China, Taiwan, Nepal and north India, epidemic patterns with seasonal outbreaks linked to increased rainfall are more typical. In Southern areas such as Indonesia, Cambodia, Southern Thailand and Timor-Leste the endemic pattern results in sporadic cases occurring throughout the year [6].

Substantial progress has been made in JE surveillance and the introduction of immunisation programmes since the 1990’s. By 2012, 75 % of countries with transmission risk were carrying out surveillance programs and 46 % had a childhood immunisation programme [4]. In countries such as Nepal, China and Thailand the incidence of JE is stabilising or decreasing as a result of these measures [2]. The World Health Organisation states ‘JE vaccination should be integrated into national immunization schedules in all areas where JE is recognized as a public health priority. Even if the number of JE-confirmed cases is low, vaccination should be considered where there is a suitable environment for JEV transmission, i.e. presence of animal reservoirs, ecological conditions supportive of virus transmission, and proximity to other countries or regions with known JEV transmission’ [1]. However it must be remembered that enzoonotic transmission will continue despite a country having its population fully vaccinated. A review of cases reported in Japan from 1992–2004 found that 361 cases were recorded, predominantly in unvaccinated older individuals [7].

JE remains, however, a rare disease in travellers. Estimates vary but an oft-quoted figure is a 1 in 1 million risk per month. One recent review analysed 21 confirmed cases in travellers reported between 1992 and 2008 [8]. Based on Swedish data the authors estimated an attack rate of 1/400 000 per trip, regardless of type or length of stay. Other commentators analysed European data and proposed an overall disease incidence of 1.3 cases per 7.1 million travellers [9]. A recent small sample of nearly 400 Australian travellers to Asia found no sero-conversion for JE– however the small numbers make this difficult to interpret [10].

Most National Vaccination Guidelines have focused on the length of travel and the time of year when assessing risk in order to provide recommendations. However an analysis of the 21 cases mentioned above showed that 70 % of individuals had travelled ‘short term’ (between 10 days and 5 weeks) and 35 % of the cases occurred outside of the ‘high risk transmission’ season. For example, of eleven cases recorded in travellers to Thailand between 1992 and 2008, six occurred outside of the monsoon season. Of note these were mainly in Southern Thailand which has a more endemic pattern of transmission.

For some years the principal vaccine available to travellers was the first-generation, mouse brain derived, inactivated Nakayama strain JE-MB (sold as ‘JE-VAX’ or ‘Biken’). This vaccine was associated with a risk of hypersensitivity reactions which occurred at a rate of between 1-17/10 000 vaccinees. More concerning was the rare but severe, and occasionally fatal, acute disseminated encephalomyelitis temporally associated with the administration of this vaccine [3]. The vaccine was recommended as a 3 dose schedule of 0, 7 and 30 days, with 100 % of recipients having neutralizing antibodies at 6 months. In a trial of boosters, immunity persisted in 21 persons at 2 years who did not receive a booster, so boosting recommendations were not clear [11]. JE-MB vaccine is no longer manufactured in the US, Europe, Japan or Australia, however a version of this, known as ‘Green Cross’ or ‘Boryung ‘from its Korean manufacturers, is still utilised in some Asian countries.

In 2009 an expert opinion on vaccination of travellers against Japanese encephalitis was published. This was in response to the disease data in travellers and the availability of newer, safer vaccines. The authors stated that, with the availability of safer vaccines ‘the focus of travel counselling on JE risks and prevention can now be directed at the risks of acquiring the disease and its consequences, instead of the risk of the vaccination’ [3].

In 2009 a Vero cell derived inactivated two dose vaccine was introduced, JE-VC. It is marketed as IXIARO® in the US and most other countries, JESPECT® in Australia and New Zealand and JEEV® in India. JE-VC vaccine is composed of proteins from inactivated JEV plus 0.1 % aluminium hydroxide adjuvant. It requires cold chain storage at 2–8 °C, protection from light, and cannot be frozen. In the approval trial 96 % of per-protocol vaccinees developed neutralizing antibody titre levels thought to be protective at 28 days after the last dose with development of protective antibodies in as little as 7 days after the second dose [11]. The approved vaccine schedule involves two doses provided 28 days apart. Side effects were mild and identical to adjuvant placebo with site reactions in <1 % and headache, myalgias, influenza-like illness and fatigue as the most common findings, occurring in 13–26 % of recipients in the first week [11]. JE-VC is now approved in the US for children from 2 months of age [12]. One dose is only partially immunogenic, 41 % of vaccinees had protective immunity at 28 days, declining to 26 % at 56 days. However, it should be noted that a single dose of JE-VC has been shown to be immunogenic as a booster in those previously primed with a full course of JE-MB [13] and thus a full vaccination history should be taken. Guidance on boosting a full JE-VC series with a JE-VC booster varies from country to country due to a lack of long term immunological data and trials and hence is beyond the scope of this paper. One recent paper however showed immunity at 6 years when a JE-VC series was boosted with a 3^rd^ JE-VC vaccination at 1 year, suggesting some durable immunity with boosting [14].

A common problem is travellers attending for consultation too late to receive two doses of JE-VC at the recommended 28-day interval. Thus a frequently posed question on the International Society of Travel Medicine listserv is – “where can my patient get their second dose of IXIARO® in Asia?” - indeed a review of listserv posts showed 75 on this subject between July 2014 and May of 2015 (http://myistm.istm.org/digestviewerdashboard/?communitykey=1d8172ae-b1cf-48fe-b828-e0bab00a2869&tab=digestviewerdashboard&AuthenticationToken=UZKETOY2-GW7S-QHCQ-B71I-YM3SYKOTOBXZ). At this stage JE-VC has only limited availability in Asia, thus, constraining options for vaccination in the destination country.

An alternative therefore might be acceleration of the vaccination schedule. This hypothesis has been successfully proven in a clinical trial in Hepatitis B vaccines [15]. The data on JE vaccines is not as robust, however there are some recently reported findings. A recent study concluded JE-VC vaccine given via an accelerated 2-dose regimen within one week induced strong immune response similar to that obtained with the conventional 28 day schedule, with a satisfactory tolerability profile. This is of course off label until published and approved by National bodies but may be considered as a possible alternative to the traditional schedule where risk of disease outweighs forgoing vaccination due to lack of time to get immunologic response per established schedules [16].

The previously quoted study suggests 41 % of adults developed antibodies to JE 28 days after a single dose of JE-VC; however this dropped to 26 % after 56 days, suggesting one dose is not an acceptable vaccination strategy for JE-VC [11].

Thus options for last minute travelers are off license rapid schedules or offering one vaccine with the understanding that it is unlikely to offer adequate protection.

In 2012, Imojev, a novel recombinant chimeric virus vaccine (CV-JE), was licensed in Australia and Thailand. CV-JE is based on 17D-204 yellow fever vaccine, with 2 genes deleted and replaced with JE pre-membrane (prM) and Envelope (E) genes from SA 14-14-2 attenuated virus [17]. It requires cold chain storage at 2–8 °C and protection from light (http://products.sanofi.com.au/vaccines/IMOJEV_AUS_PI.pdf). The vaccine expresses prM and E proteins which are antigenic and stimulate neutralizing antibodies and cell mediated immunity. Neutralizing antibodies were present in 89 % of vaccinees 28 days after vaccination and persisted in 87 % of vaccinees at 60 months after vaccination (http://products.sanofi.com.au/vaccines/IMOJEV_AUS_PI.pdf) [18]. This single dose live vaccine is highly immunogenic (equivalent to 3 dose inactivated JE-MB) and is currently the preferred vaccine in Australia due to its longer predicted period of protection and ease of administration. It should be noted that this is a live vaccine and as such is contraindicated in the immunosuppressed, and pregnant or breastfeeding women. It is licensed from 12 months of age onwards and a booster is recommended after 1–2 years for children under 18 who have not received previous vaccination with JE-MB or JE-VC (http://products.sanofi.com.au/vaccines/IMOJEV_AUS_PI.pdf). Most common side effects reported in the approval trial were headache, fatigue, myalgias and malaise ranging from 17–24 % of vaccinees. Vaccine site reactions occurred in 12 % of recipients (http://products.sanofi.com.au/vaccines/IMOJEV_AUS_PI.pdf). CV-JE is currently licensed in Australia, Thailand, Malaysia, the Philippines, Hong Kong, and Singapore and can be found in expatriate clinics in a number of other countries in the region. Receiving the CV-JE vaccination in country could be an attractive option for some travellers, with awareness that it will take at least fourteen days to achieve an adequate level of protection.

Japanese Encephalitis Vaccine, Live (JE-LV) (‘Chengdu’ for short after the name of its Chinese manufacturer) is a live attenuated JE vaccine consisting of SA 14-14-2 attenuated virus, grown in hamster kidney cell culture. It requires cold chain storage at 2–8 °C and protection from light. This is also a single dose live vaccine and as such must be given 28 days after other recent live virus vaccines such as measles or zoster. Neutralizing antibodies are generated in 90 % of vaccinees according to the WHO guideline (http://www.who.int/immunization_standards/vaccine_quality/pq270_vpsar_jevlive_chengdu_12nov13.pdf). With one study showing 5 year protective immunity of 96 % in a follow-on cohort in Nepal [19].

Thus taking into account the availability of these newer and safer vaccines the advice of the Expert Committee comprised two categories, either advise vaccine or consider vaccine [3].

The ‘Advise’ category reads as follows: Providers should advise all travellers to areas of Asia where JE virus is transmitted in an enzootic cycle (including Japan) about the risks and consequences of JE and characteristics of available vaccines.

JE vaccine is recommended for:

- All expatriates
- Repeat travellers who return frequently to the region or who, cumulatively, have a prolonged duration of exposure.
- Any individual with a prolonged duration of stay, independent of itinerary.
- Any individual with a travel itinerary including rural areas.
- Travellers wishing maximum protection

However, it fails to take into account the necessary time required to administer the complete vaccine series, and failure of this information to be widely known at the level of the general public who are travelling so they can prepare adequately before their departure, is a common occurrence in our experience.

The ‘Consider’ vaccination category reads as follows: Providers should consider vaccinating the following types of patients

- All other travellers visiting regions with enzootic transmission during a transmission period, particularly:
  - Those with greater outdoors exposure
  - Individuals > 50 years of age
  - Children < 10 years of age
  - History of chronic conditions such as
  - History of solid organ transplant
  - Cochlear implants, ventriculoperitoneal shunts and other devices impinging on the central nervous system
  - Hypertension
  - Diabetes mellitus
  - Chronic renal disease
  - Anti-TNF therapy
  - Pregnant women (balancing unknown risks associated with vaccine)
  - Individuals known from previous history to be homozygous for CCR5Delta32 (which has been shown to increase the risk of symptomatic West Nile infections) [2]

The recommendations for the second group take into account host factors that may put individuals more at risk and do not rely solely on length or season of travel. Most national immunisation recommendations are less detailed than this.

## International Country Guidelines for the Administration of JE Vaccine

A review of the readily accessible international guidelines revealed the following guidance, (note: all these countries currently use JE-VC vaccine except Australia where CV-JE vaccine is also available):

### WHO

- JE vaccination is recommended for travellers to endemic areas with extensive outdoor exposure during the transmission season [1]

#### Australia

JE vaccination is recommended for

- Travellers (≥12 months of age) spending 1 month or more in rural areas of high-risk countries in Asia and Papua New Guinea, however, as JE has occurred in travelers after shorter periods of travel, JE vaccination should be considered for shorter-term travelers, particularly if the travel is during the wet season, or anticipated to be repeated, and/or there is considerable outdoor activity, and/or the accommodation is not mosquito-proof
- All other travellers spending a year or more in Asia (except Singapore), even if much of the stay is in urban areas.

All travellers to Asia (and other tropical regions) must be fully aware of the need to take appropriate measures to avoid mosquito bites. The risk of JE to travellers to Asia is determined by the season of travel, the regions visited, the duration of travel, the extent of outdoor activity and the extent to which mosquito avoidance measures are taken. Clearly the risk is greater during prolonged travel to rural areas of Asia during the wet season; it is probably negligible during short business trips to urban areas.

There are also specific recomendations for the Torres Strait Islands as follows: JE vaccination is recommended for

- All residents (≥12 months of age) of the outer islands in the Torres Strait
- All non-residents (≥12 months of age) who will be living or working on the outer islands of the Torres Strait for a cumulative total of 30 days or more during the wet season (December to May). Those visiting only the inner islands, including Thursday Island, do not require vaccination [20].

### United States Centres for Disease Control

- JE vaccine is **recommended** for travelers who plan to spend 1 month or more in endemic areas during the JE virus transmission season. This includes long-term travelers, recurrent travelers, or expatriates who will be based in urban areas but are likely to visit endemic rural or agricultural areas during a high-risk period of JE virus transmission.
- Vaccine should also be **considered** for the following:
  - Short-term (less than1 month) travelers to endemic areas during the transmission season, if they plan to travel outside an urban area and their activities will increase the risk of JE virus exposure. Examples of higher-risk activities or itineraries include: 1) spending substantial time outdoors in rural or agricultural areas, especially during the evening or night; 2) participating in extensive outdoor activities (such as camping, hiking, trekking, biking, fishing, hunting, or farming); and 3) staying in accommodations without air conditioning, screens, or bed nets.
  - Travelers to an area with an ongoing JE outbreak.
  - Travelers to endemic areas who are uncertain of specific destinations, activities, or duration of travel.

JE vaccine is **not recommended** for short-term travelers whose visits will be restricted to urban areas or times outside a well-defined JE virus transmission season (www.cdc.gov).

### United Kingdom “Green Book’

Travellers to South and South-East Asia and the Far East should be immunised if staying for a month or longer in endemic areas during the transmission season, especially if travel will include rural areas. Other travellers with shorter exposure periods should be immunised if the risk is considered sufficient. For example, those spending a short period of time in rice fields (where the mosquito vector breeds) or close to pig farming (a reservoir host for the virus) should be considered for vaccination [21].

### Canada

Generally, IXIARO® is recommended for travel to JE endemic/epidemic areas during the risk season:

- For all travellers who spend more than a cumulative total of 30 days in rural areas (or in urban areas known to be endemic or epidemic for JE); this might include longer term travellers or expatriates who, while based in urban areas, might be expected to make intermittent short trips to rural areas of risk;
- For travellers who will spend less than a cumulative total of 30 days in rural areas(or in urban areas known to be endemic or epidemic for JE) if they expect to have substantial cumulative activity outdoors (or indoors if the indoor area does not exclude mosquitoes), especially during the evening/night.

Generally, IXIARO® use is not recommended for JE endemic/epidemic areas during the risk season:

- For travellers whose entire itinerary will be in urban areas (unless the urban areas are known to be endemic or epidemic for JE);
- For travellers whose visits to rural areas (or urban areas known to be endemic or epidemic for JE) will be during the daytime only (http://www.phac-aspc.gc.ca/publicat/ccdr-rmtc/08vol34/acs-4/index-eng.php).

European Guidelines were not readily accessible to the authors and as a result an email and telephone survey of Eurotrop Members was conducted to elucidate the existence of other national guidelines. The following responses were provided:

### Spain

National recommendations on Japanese Encephalitis vaccination in travellers exist. However, it is a very “open” recommendation: Vaccine is recommended for those travellers with long exposure (cyclist, occupational exposure, camping…) in risk areas and during seasonal transmission, especially in areas with risk of floods.

### Czech republic

No national recommendations on Japanese Encephalitis vaccination in travellers exist. However, vaccination is recommended for travellers with more than four weeks stay in areas with transmission of JE (some areas only in the rainy season), especially if they stay in rural areas. The respondent acknowledged awareness of vaccine manufacturer recommendation, and that local practices differ from vaccine indications.

### Poland

No national recommendations on Japanese Encephalitis vaccination in travellers exist.

The vaccination is not available in the country, and it requires a special import procedure from a manufacturer signed by the Ministry of Health. The respondent’s institutional policy recommends vaccination for long stays in endemic regions of at least 4 weeks duration with excursions to rural areas especially missionaries, soldiers, round the world travelers, and hunters.

### Denmark

National recommendations on Japanese Encephalitis vaccination in travellers exist, vaccination is recommended for travellers with more than four weeks stay in areas with transmission of JE (some areas only in the rainy season), especially if they stay in rural areas.

### Italy

No national recommendations on Japanese Encephalitis vaccination in travellers exist. The respondent’s institutional policy recommended vaccination for travel in endemic regions *and* stay in a rural setting for 1 month or more including if a seasonal pattern is well recognized.

### Portugal

No national recommendations on Japanese Encephalitis vaccination in travellers exist. The respondent’s institutional policy recommended to follow WHO / CDC / Health Canada recommendations, i.e. Vaccination is recommended for travellers with more than four weeks stay in areas with transmission of JE (some areas only in the rainy season), especially if they stay in rural areas.

### Availability of Vaccine in Asia as of May 2015

Clinics in Asian countries listed on the International Society of Travel Medicine website, and other clinics known to the authors as providing care to expatriates and travellers in Asia, were contacted via email regarding the availability of each type of vaccine (JE-MB ‘Green Cross’, JE-VC ‘Ixiaro/Jespect’, CV-JE ‘Imojev’ and JE-LV ‘Chengdu’) in their location. The results of reported vaccine availability in certain locations follow in Table 1 and below; these findings do not serve as an endorsement for these facilities or providers. Other references include the Travel Medicine directory of the International Society of Travel Medicine’s website http://www.istm.org/AF_CstmClinicDirectory.asp.

**Table 1:** List of Japanese Encephalitis vaccines by country

Country	Vaccines Available	Vaccine Licensed?	Cities where available
Bangladesh	JE-VC		Dhaka
Bhutan	None		
Brunei	CV-JE	CV-JE	Multiple
Cambodia	CV-JE, JE-LV		Pnomh Penh
China	JE-LV		Beijing
Hong Kong	JE-VC, CV-JE	JE-VC, CV-JE	
India	JE-VC		Multiple
Indonesia	JE-MB, some CV-JE		Jakarta, Denpasar
Japan	JE-VC	JE-VC	Multiple
Laos	Some CV-JE (expats only)		Vientiane
Malaysia	CV-JE	CV-JE	Multiple
Myanmar	CV-JE		Yangon
Nepal	JE-LV		Kathmandu
Pakistan	None		
Papua New Guinea	Some JE-VC (expats only)		Port Moresby
Philippines	CV-JE	CV-JE	Multiple
Singapore	JE-VC, and CV-JE		
South Korea	JE-MB		Multiple
Thailand	CV-JE		Multiple
Timor Leste	JE-VC		Dili
Vietnam	JE-MB some		Hanoi, Ho Chi Minh City
	CV-JE		

Key: JE-VC Vero cell Vaccine (Ixiaro, Jespect, JEEV); CV-JE Chimeric Vaccine (Imojev); JE-MB Mouse brain vaccine (Green Cross); JE-LV Live attenuated vaccine (Chengdu)

## Conclusions

At least 9 countries now recommend vaccination for a substantial number of travelers to Asia. These guidelines generally recommend vaccination with JE-VC or CV-JE. Unfortunately JE-VC, the most commonly available vaccine in North America, Europe and much of the world requires multiple vaccinations over a month and may not be suited to last minute travelers. Furthermore, JE-VC has limited availability in Asia and completing the course during travel is a logistically difficult option. Many Asian countries stock alternate vaccines including CV-JE, JE-MB or JE-LV, which are currently unavailable to many travelers pre-departure.

Travel clinic providers have options including

- Use of an ‘off-label’ accelerated vaccine schedule with the tradeoff of possible loss of durable immunity requiring boosters in the future.
- Contacting one of the few expatriate clinics that stock JE-VC in Asia and completing the series there.
- Receiving CV-JE or JE-LV in Asia as a 1 shot vaccine with high and durable immunity that may be delayed for several weeks after vaccination (ensuring no contraindications to live vaccine administration)

The last 2 require amendments to the traveler’s itinerary and obviously are not well suited to a short vacation; however this type of traveler is at relatively low risk of acquiring JEV. A traveler with a longer itinerary who is at higher risk of exposure to JEV should have more flexibility in their ability to go get vaccinated whilst in Asia.
